# Mound Builders

**Published:** 1893-03

**Authors:** 


### MOUND BUILDERS.

A skeleton was recently discovered in a mound upon a farm near Frankfort, Ohio. This mound was oval in shape, with diameters of one hundred and ten feet and sixty feet. Its original height was, apparently, about ten feet, but from various causes has been reduced to five feet. Its structure was more or less stratified, and consisted of successive layers of loam, sand, and yellow clay. Several skeletons were found in this mound, but with one exception they were almost entirely decomposed, and crumbled away into dust on exposure to the air. One was that of a man over six feet in height, and the size of the bones showed him to have possessed superior strength. Near the head were found five teeth of a bear and two of a panther. These were all perforated, and doubtless formed a necklace or amulet. On the right side of the skeleton was found a plate of copper measuring six by seven inches; on the left were several articles consisting of two disks of copper joined together by a cylinder of the same metal, forming a spool-shaped body. These objects are very abundant in mounds, and are considered by Professor Putnam to have been used as ornaments and suspended from the ears by cords, traces of which occasionally still remain. A large number of shells was found near the skeleton.

The other skeletons found in this mound showed a remarkable diversity in the method of burial; the greater part of them were interred lying at length; some, however, were placed in a contracted position, the knees being bent up towards the chin. Two among them had evidently been burnt either before or after death. These different forms of sepulture may indicate burials at different ages or by different races, or, more likely, a difference in the rank of those buried; and the presence of the charred human bones points strongly to the existence of sanguinary funeral rites, such as in some savage tribes of the present day necessitate the sacrifice of human beings to accompany their chief on his journey to "the undiscovered country." The same mixture of buried and burnt bodies has been noticed in certain tumuli in Brittany.

Among other discoveries of the above named explorers was one of the so-called "altars" of burnt clay, which are frequently found in the mounds. It was rectangular in form, with symmetrically rounded corners, and measured thirty by twenty-four inches. In the centre was an oval depression measuring eighteen by twelve inches and four inches in depth. This depression contained ashes and fragments of human bones. Various objects have been found upon these altars, but all bear marks of the action of fire, and were undoubtedly connected with religious or funeral rites.

Among other objects found in the vicinity of the Porter mound was the shoulder blade of some large mammalian animal, more than six inches in length, and pierced with two holes, so that it could be suspended like the gorgets worn by Indians of recent times. There were also found nearly a thousand small pearls, a plate of copper carefully wrapped in cloth, flint hatchets, knives, and arrowheads, and pieces of pottery; but all the discoveries hitherto made throw but little light upon those mysterious people, who, for want of a better name, we call the Mound-builders. While they are apparently distinct from the Indians who occupied the continent at the time of its discovery, they may have flourished in comparatively modern times, perhaps not many years before the coming of Columbus. Their civilization resembles in many respects that of the men of the bronze age in Europe, but with the notable exception that native copper was the only metal used by them. This is found quite abundantly in North America, while the men of the bronze age in Europe seem to have been equally familiar with both metals from the time of their first appearance, and possessed the art of smelting them together into bronze. The American Mound-builders, on the contrary, were only acquainted with copper, and only knew how to hammer it out while cold, and were ignorant of the art of melting and casting. They seem to have been a somewhat settled race, carrying on an imperfect system of agriculture, but extremely warlike, as their forts, battle-fields, and burial places in the Ohio valley indicate. But whence they came, whither they disappeared, or what relation they bore to other races, both on the American and other continents, or the date at which they flourished in the great central valley of the United States, are questions to which at present no satisfactory answer can be given, and to which it seems very improbable that any answer will ever be found.

